# Inoculation with *Azospirillum brasilense* Associated with Nitrogen Rates on the Yield and Nutritional Value of Giant Sorghum Silage

**DOI:** 10.3390/ani16040557

**Published:** 2026-02-11

**Authors:** Luciely Bordallo da Conceição Chagas, Sheila Vilarindo de Sousa, Mariane Alves da Silva, Julián Andrés Castillo Vargas, Perlon Maia dos Santos, Daniel Rume Casagrande, Daiane de Cinque Mariano, Ricardo Shigueru Okumura, Raylon Pereira Maciel

**Affiliations:** 1 Campus of Parauapebas, University Federal Rural of the Amazon, Parauapebas 68515-000, Brazil; luciely.chagas@parauapebas.pa.gov.br (L.B.d.C.C.); mariane.alves.silva@gmail.com (M.A.d.S.); perlon@ufra.edu.br (P.M.d.S.); 2 Postgraduate Program in Tropical Animal Science, University Federal of Piaui, Teresina 64049-550, Brazil; sheila.vilarindo@ufra.edu.br; 3 Center for Agricultural and Biological Sciences, State University of Vale do Acaraú, Acaraú 62580-000, Brazil; julian_vargas@uvanet.br; 4 Department of Animal Science, University Federal of Lavras, Lavras 37200-000, Brazil; danielcasagrande@ufla.br; 5 Department of Soils and Rural Engineering, University Federal of Mato Grosso, Cuiabá 78060-900, Brazil; daiane.mariano@ufmt.br (D.d.C.M.); ricardo.okumura@ufmt.br (R.S.O.)

**Keywords:** plant growth-promoting bacteria, fermentation parameter, bolivian giant sorghum, forage yield, total digestible nutrients

## Abstract

The high cost of nitrogen (N) fertilizers has increased interest in sustainable alternatives, such as inoculation with *Azospirillum brasilense*, to improve forage production. This study evaluated the agronomic performance, yield, nutritional composition, and fermentation profile of giant sorghum silage from crops inoculated with *A. brasilense* under different N rates. A randomized block design was adopted in a 5 × 2 factorial arrangement, with five N rates (0, 50, 100, 200, and 400 kg ha^−1^) and the presence or absence of *A. brasilense* inoculation, with four replicates. Nitrogen rates significantly affected plant height and stem diameter, with maximum values observed at 200 kg ha^−1^ N. An interaction between inoculation and N rates was detected for first-cycle yield, in which inoculated plants showed reduced productivity at 200 and 400 kg ha^−1^ N. Total yield was higher in non-inoculated treatments and was maximized at 200 kg ha^−1^ N, representing a 29.42% increase compared with the unfertilized control. Crude protein concentration increased with increasing N rates, while in vitro dry matter digestibility was influenced only by N fertilization. Inoculation did not improve agronomic performance and negatively affected yield at higher N rates, although both inoculation and N fertilization influenced protein and fiber contents of sorghum silage. Under the edaphoclimatic conditions of the Brazilian Amazon, *A. brasilense* inoculation is not recommended for giant sorghum cultivation, whereas the application of 200 kg ha^−1^ N was the most effective strategy to maximize yield.

## Simple Summary

This study evaluated the effect of inoculating with the bacterium *Azospirillum brasilense*, in conjunction with nitrogen side-dressing. By assessing the agronomic and productive characteristics of the plant and the nutritional value of the silage, we found that inoculation with *A. brasilense* did not improve the agronomic characteristics of forage sorghum, and resulted in decreased productivity at higher N rates. Although inoculation slightly increased silage crude protein concentration, this effect was insufficient to compensate for yield losses or to reduce the need for mineral N. For forage management, a topdressing application of 200 kg ha^−1^ of N is recommended, without seed treatment with the bacterium. These findings shed light on the responses of giant sorghum inoculated with the bacterium *A. brasilense* and highlight the importance of understanding and further investigating the interactions of bacteria in giant sorghum seeds under the soil and climate conditions of the Brazilian Amazon.

## 1. Introduction

Silage is a widely used practice in animal production to ensure quality feed during periods of scarcity [1]. Corn is the main forage species used worldwide [2] due to the adequate levels of water-soluble carbohydrates found in the plant, which lead to lactic fermentation, promoting the preservation of a feed with high nutritional value, easy to prepare and highly accepted by animals, with high green mass yield and adequate dry matter content [3].

However, despite being the most important crop for agricultural production, it is more sensitive to water deficit than other grasses [4]. In tropical regions, particularly in Brazil, sorghum as a drought-tolerant, low-fertility-adapted, high-yielding alternative to corn for silage [5,6,7]. Additionally, the sorghum plant stands out for being a feed with high nutritional value, a high concentration of water-soluble carbohydrates, essential for adequate lactic fermentation, and for its high dry matter yields per unit area [8,9], which can be similar to silages made from corn plants [10].

Among the forage sorghum options available on the market, great emphasis has been placed on those of tall, high-yielding genotypes, known as Bolivian giant sorghum [11,12]. Despite its productivity, it typically has a low or no grain content, which compromises its nutritional value [11]; this could be improved with the appropriate use of fertilizers [13].

Of the essential nutrients for sorghum development, nitrogen is the most demanding [14] because it affects their structural characteristics, which is reflected in digestive behavior (due to forage availability) and animal production per unit area [15]. The absence of nitrogen (N) leads to a reduction in production due to the impairment of protein and pigment synthesis in plant tissues related to photosynthesis [16].

Nitrogen fertilization is among the agricultural practices with the highest investment costs in agricultural production [17], resulting in losses due to NH_3_ volatilization [18], and N_2_O emissions [19]. Ammonia (NH_3_) is not a direct greenhouse gas, as it can be deposited in the atmosphere and return to the soil, thereby feeding the nitrification and denitrification cycles, which result in indirect N_2_O emissions [20]. As an alternative to provide N to plants and support sustainable production, inoculation of plants with plant growth-promoting bacteria has been recommended [21,22], with results from *Azospirillum brasilense* for forage species [23,24].

*A. brasilense*, a plant growth-promoting bacterium, improves the synthesis of phytohormones (cytokinins, indoleacetic acid, and gibberellins), increasing nitrogen availability, and boosting dry matter production in forages in a less costly and sustainable manner [25,26].

In sorghum, inoculation with *A. brasilense* has been shown to modify root growth [27], increasing total dry mass for silage production [28,29]; however, there is no information on the performance and silage quality of giant sorghum inoculated with *A. brasilense* under the soil and climate conditions of the Brazilian Amazon.

The research hypothesis is that inoculation with AbV5 and AbV6 strains promotes partial fulfillment of the plant’s nitrogen (N) needs. This study is crucial as it aims to evaluate the inoculation of the bacterium *A. brasilense*, associated with nitrogen fertilization, on the agronomic and productive characteristics, as well as the nutritional value of giant sorghum cultivated in the Brazilian Amazon. The findings of this research could significantly contribute to the understanding and improvement of sorghum cultivation in tropical conditions, making it a research of high importance and interest to your audience.

## 2. Materials and Methods

### 2.1. Location and Characterization of the Area

The experiment was conducted under field conditions in the experimental area (06°04′16.4″ S and 49°08′8.3″ W, 270 m) of the Forage Sector of the Federal Rural University of the Amazon, Parauapebas city, Brazil, from February to June 2021. A toposequence slope with gentle relief characterizes the experimental area, and the soil is classified as sandy loam. The chemical characteristics of the soil, in the 0–20 cm layer, were: pH 5.5, P = 0.32 mg dm^−3^, K = 28.24 mg dm^−3^, Ca = 0.90 cmol_c_ dm^−3^, Mg = 0.32 cmol_c_ dm^−3^, Al = 0.50 cmol_c_ dm^−3^, H + Al = 5.80 cmol_c_ dm^−3^, CEC pH7 = 7.10, and OM = 1.40 g dm^−3^.

**Figure 1:**
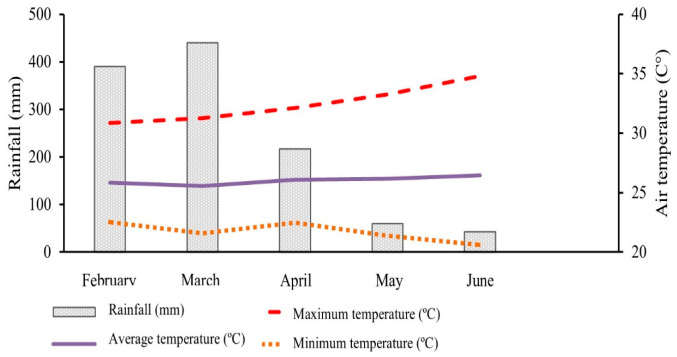
Meteorological data collected from the National Institute of Meteorology (INMET) during the cultivation of forage sorghum AGRI 002E (February to June 2021) in Parauapebas city, Brazil.

The climate is classified as Aw, a tropical rainy type, with rainfall concentrated in summer and a dry season in winter, and can vary to Aw’, with summer and autumn rains. The rainfall pattern, average relative humidity, and temperature (minimum, average, and maximum) associated with the sorghum production cycle are illustrated in Figure 1.

The plots (4.2 × 5.0 m) consisted of 6 rows with a spacing of 0.7 m between rows and 0.2 m between plants, totaling a population density of 71,428.5714 plants ha^−1^.

### 2.2. Experimental Design, Azospirillum Strains, and Inoculation Procedure

The soil was prepared by plowing and harrowing, followed by the application of 3.28 t ha^−1^ of dolomitic limestone. Fertilization at sowing was carried out with 989 kg ha^−1^ of single superphosphate (18% P_2_O_5_) and 451 kg ha^−1^ of potassium chloride (60% K_2_O), as recommended by Sousa and Lobato [30].

The giant sorghum used was the AGRI 002E hybrid, planted on 27 February 2021. The seeds were inoculated using the liquid inoculant AzoTotal^®^ obtained from the Total Biotecnologia company, Curitiba, Brazil, contained a concentration of 2 × 10^8^ CFU mL^−1^ of the AbV5 (=CNPSo 2083) and AbV6 (=CNPSo 2084) strains, at a dose of 200 mL of inoculant in 25 kg of seeds, after a brief period of drying in the shade, sowing was conducted according to Silva et al. [31].

The experiment was arranged in a randomized complete block design in a 5 × 2 factorial scheme. This design was chosen to study the combined effects of five N rates for topdressing nitrogen (0, 50, 100, 200, and 400 kg ha^−1^ of N) and the presence or absence of seed inoculation with *A. brasilense*. The experiment had four replicates, and topdressing N applications were made at 30, 55, and 80 days after emergence.

### 2.3. Data Collection

The agronomic characteristics of giant sorghum were evaluated at harvest, 97 days after emergence. They were specified as follows: plant height (PH), distance between the plant collar (base of the plant close to the ground) and the insertion of the last leaf, measured with a measuring tape; and stem diameter (SD), measured at 0.10 m above ground level using a digital caliper [32]. Sorghum yield was estimated by harvesting the two central rows, followed by weighing on a digital scale, and was expressed in tons per hectare. First-cycle yield (FiYield), regrowth yield (ReYield), and total yield (Yield) were calculated, representing the sum of FYield and RYield. These terms refer to specific aspects of the sorghum yield and are important for understanding the overall yield of the crop [33].

### 2.4. Harvest and Silage Production

Giant sorghum was harvested when the plant reached 30–35% dry matter content, 97 days after emergence, with a cutting height of 15 cm from the ground. The material was chopped using a tractor-mounted silage harvester and subsequently placed in experimental polypropylene bucket silos, closed with lids, fitted with a Bunsen valve, and sealed with adhesive tape [34]. Regrowth was harvested 153 days after the first cut, and no top dressing was applied.

### 2.5. Chemical Composition Analysis

After 139 days of ensiling, the silos were opened and the deteriorated parts discarded. The material was homogenized and then sampled for subsequent chemical analysis. To determine pH, 10 g of fresh silage were added to 90 mL of distilled water. After 60 min, readings were taken with a portable digital potentiometer in triplicate, with the average of the three measurements considered, as described by Bolsen et al. [35].

Ammonia nitrogen (N-NH_3_) was determined by colorimetry, using the methodology described by Chaney and Marbach [36], adapted for silage samples. A total of 12.5 g of green silage samples were placed in plastic containers with lids and added to 100 mL of 0.2N H_2_SO_4_ solution. After resting for 48 h, the samples were filtered, and 1.5 mL aliquots of the extract were removed and placed in two 2.0 mL Eppendorf tubes. The material was then centrifuged for 10 min at 13,000 rpm. Aliquots were removed and stored in other Eppendorf tubes, which were kept in a freezer at −20 °C until analysis. To read the samples, a calibration curve was prepared using ammonium chloride at a volume of 0, 5, 10, 15, 20, and 25 μL. Then, homogenization was performed, and the mixture was incubated in a water bath at 39 °C for 15 min. After this period, the reading was performed on a spectrophotometer using a wavelength of 630 nm. The absorbance data from the calibration curve were used to construct the regression curve, thus allowing the determination of the ammonia nitrogen concentrations of the samples.

The sorghum samples, both before ensiling and in silage, were pre-dried in a forced-air oven at 55 °C for 72 h and subsequently milled in a Willey mill using a 1 mm sieve. Dry matter (DM—method G-0003/1), mineral matter (MM—method M-001/2), and crude protein (CP—method N-001/2) were determined using the methodology described by Detmann et al. [37]. Neutral detergent fiber (NDF) and acid detergent fiber (ADF) were determined according to Van Soest et al. [38], with modifications as proposed in the Ankom device manual (Ankom 200, Technology Corporation, Macedon, NY, USA), using thermostable alpha-amylase.

Total digestible nutrient (TDN) values were measured using the equation proposed by Rodrigues [39]:

TDN: 87.84−(0.7×%ADF)

### 2.6. Evaluation of Organic Acids

For the analysis of organic acids (acetic, butyric, and lactic), 10 g of wet silage sample were diluted in 90 mL of Milli-Q water. Subsequently, the material was subjected to maceration for 1 min and then filtered. From the material obtained, 2 mL of the filtrate were pipetted and subsequently added to 1 mL of metaphosphoric acid solution (20% *w*/*v*). The mixture was then acidified with 50 microliters of sulfuric acid (H_2_SO_4_) at 50% (vol/vol). Then, the material was subjected to homogenization and transferred to a 2 mL Eppendorf tube. Afterward, it was centrifuged at 13,000 rpm for 10 min. Subsequently, the supernatant was transferred to other Eppendorf tubes, which were identified and kept in a freezer at −20 °C. Subsequently, the samples were analyzed by high-performance liquid chromatography (HPLC-DAD, Shimadzu, Tokyo, Japan) according to the chromatographic conditions suggested by Vargas et al. [40].

### 2.7. Digestibility

To assess in vitro dry matter digestibility (IVDMD), we followed the method proposed by Tilley and Terry [41] and adapted by Silva et al. [42] meticulously. We used penicillin vials and collected ruminal fluid from a fistulated steer fed a diet with a 40:60 forage/concentrate ratio and 15.3% crude protein. The ruminal fluid was immediately transferred to a preheated thermos flask at 39 °C, and the homogenized material was filtered through four layers of gauze. We used a buffer solution proposed by McDougall [43] and added 5 mL of urea solution (5.5 g/100 mL) for every 300 mL of buffer solution. The pH of the solution was then reduced to 6.80 by bubbling with CO_2_.

Approximately 500 mg of air-dried samples were weighed in duplicate for each sample and placed in 100 mL penicillin-type vials. Subsequently, 10 mL of ruminal inoculum and 40 mL of buffer solution (1:4 inoculum to buffer solution ratio) were added. The headspace of the vials was immediately saturated with CO_2_, and the vials were closed with rubber stoppers and aluminum seals. The vials were stored in a forced circulation oven at 39 °C. For each batch, two blank vials were evaluated. The gas produced in the vials was removed with a needle every three hours for the first 12 h and every six hours thereafter. After 48 h of incubation, the vials were opened, and the contents were transferred to filter crucibles (coarse porosity) using distilled water (temperature above 90 °C). Then, the crucibles were dried (105 °C/24 h) and weighed, obtaining the apparently undigested residue of the dry matter.

To assess in vitro neutral detergent fiber digestibility (IVNDFD), the crucibles containing the incubation residue were placed inside autoclavable universal collectors (120 mL), adding 80 mL of a neutral detergent solution, prepared according to [44], without sodium sulfite, and 250 μL of thermostable α-amylase (Termamyl 2X, Araucaria, Brazil). The collectors with the crucibles inside were closed with their respective lids and autoclaved (105 °C for one hour) according to the method described by Detmann et al. [37]. After being removed from the autoclave, the crucibles were rewashed with hot distilled water and, finally, with 30 mL of acetone. They were then dried (105 °C for 24 h) and weighed to obtain the NDF residue.

### 2.8. Statistical Analysis

The experimental data underwent a comprehensive statistical analysis, including the Shapiro–Wilk test (*p* > 0.01) and the Levene test (*p* > 0.01) to verify residual normality and homoscedasticity, respectively. The results were then subjected to analysis of variance, considering N rates, the presence or absence of *A. brasilense*, and the interaction between the two factors as sources of variation. This thorough analysis ensures the validity of our conclusions.

$$Y_{IJK}=\mu +B_{K}+E_{J}+D_{I}+{\left(B.D.\right)}_{IJ}+E_{IJK}$$

where

*Y_IJK_* = Observed value of the kth experimental unit that received treatment *I*;

*µ* = overall mean;

*B_K_* = block effect;

*E_J_* = effect of the *J*th nitrogen fertilization on the response variable;

*D_I_* = effect of the *L*th inoculation (presence or absence of *A. brasilense*) on the response variable;

(*B.D.*)*_IJ_* = effect of the interaction between the factors;

*E_IJK_* = effect of the residual random error.

When significant effects were verified, the Tukey mean test was used at a 5% probability level, using the Sisvar software version 5.3 [45].

## 3. Results

When analyzing the agronomic characteristics of giant sorghum, no interaction (*p* > 0.05) was observed between inoculation with *A. brasilense* and nitrogen fertilization for plant height, stem diameter, regrowth yield, and total yield (Table 1).

Plant height was significantly higher (*p* < 0.05) at the 200 kg ha^−1^ of N dose compared to the control treatment (without N fertilization), with values of 3.30 m and 2.96 m, respectively. This suggests that nitrogen fertilization at this dose can significantly increase plant height. Meanwhile, nitrogen fertilization at a dose of 50 kg ha^−1^ of N promoted a greater stem diameter (*p* < 0.05) in giant sorghum plants, representing an increase of approximately 19.16% at the 200 kg ha^−1^ N dose compared to the unfertilized treatment. This indicates that even a lower dose of nitrogen can have a significant impact on stem diameter.

**Figure 2:**
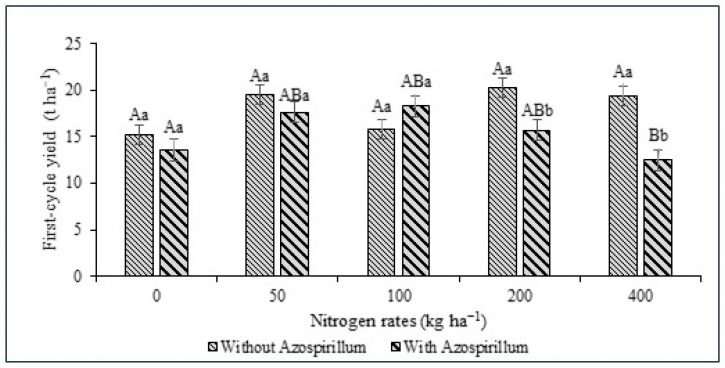
First-cycle yield of forage sorghum AGRI 002E (t ha^−1^) without and with *A. brasilense* associated with nitrogen rates (kg ha^−1^). Lowercase letters used in the results indicate differences between treatments with and without *A. brasilense* at each fertilizer dose. Uppercase letters indicate differences between nitrogen rates with and without inoculation, providing a clear interpretation of the results.

An interaction (*p* < 0.05) was observed between inoculation with *A. brasilense* and N rates on yield in the first cycle. At the 200 and 400 kg ha^−1^ N rates, the first-cycle yield showed lower values of 15.66 and 12.44 t ha^−1^, respectively, in giant sorghum plants inoculated with the bacterium (Figure 2). The interaction between inoculation and N rates resulted in reduced productivity, except at the 100 kg ha^−1^ of N dose (18.24 t ha^−1^).

While the regrowth yield variable showed no significant influence from the treatments, the total yield of giant sorghum was notably affected by the N rates (*p* < 0.05). This underscores the pivotal role of N rates, with the 200 kg ha^−1^ of N dose yielding the highest result, 29.42% higher than the unfertilized treatment. The total yield values of sorghum were lower (*p* < 0.05) in the treatments with *A. brasilense* inoculation (19.24 t ha^−1^), 13.0% lower compared to the absence of inoculation (22.12 t ha^−1^).

No statistically significant differences (*p* > 0.05) were observed for dry matter (DM), neutral detergent fiber (NDF), and acid detergent fiber (ADF) contents for seed inoculation and N rates (Table 2).The crude protein (CP) content of giant sorghum was significantly influenced (*p* < 0.05) by N rates, with silages produced from 100, 200, and 400 kg ha^−1^ of N containing 7.08%, 8.12%, and 8.76% CP, respectively.

No interaction was observed between Azospirillum inoculation and nitrogen rates for any giant sorghum silage variable (Table 3). An effect of the N dose factor (*p* < 0.05) was observed on the dry matter (DM) content of the silage, in which the dose of 100 kg ha^−1^ of N provided the highest DM content (32.09%) compared to the treatment without N fertilization (28.57%). The crude protein content of the silage was influenced (*p* < 0.05) by the isolated factors of inoculation with *A. brasilense* and N rates, verifying that the silage from sorghum inoculated with *A. brasilense* presented a higher crude protein content (7.67%) compared to the silage without the inoculant (7.01%). The rates of 200 and 400 kg ha^−1^ of N provided silages with higher crude protein contents, being 8.39% and 9.19%, respectively. The neutral detergent fiber (NDF) and acid detergent fiber (ADF) of the silages differed statistically (*p* < 0.05) with the use of the inoculant, verifying that the presence of *Azospirillum* promoted values of 70.79 and 46.54%, respectively, and in the absence of inoculation, values of 68.20% (NDF) and 44.06% (ADF) were observed.

Total digestible nutrients were not influenced by the treatments (*p* > 0.05), with values ranging from 58.89 to 60.86%. An effect (*p* < 0.05) of N rates in vitro dry matter digestibility (IVDMD) was observed, which was higher (*p* < 0.05) at the 100 kg ha^−1^ N dose (622.38 g kg^−1^) and lower compared to the 400 kg ha^−1^ N dose (547.77 g kg^−1^). In vitro digestibility of neutral detergent insoluble fiber did not differ statistically (*p* > 0.05) between treatments, with values ranging from 358.06 to 401.59 g kg^−1^.

**Figure 3:**
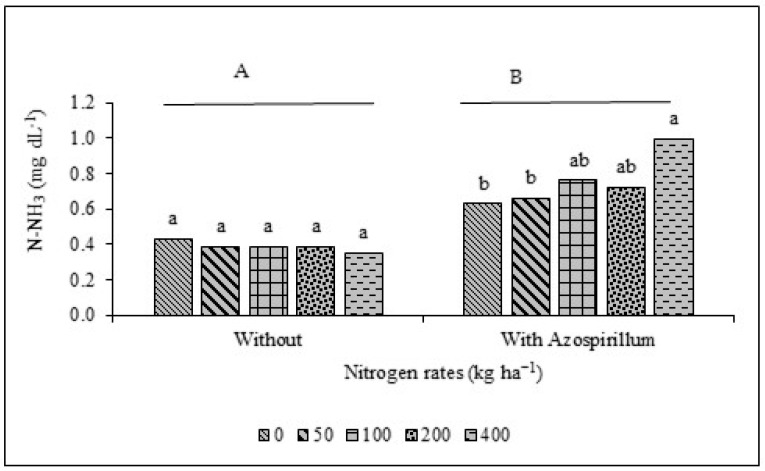
Average ammoniacal nitrogen (N-NH_3_) of forage sorghum silage AGRI 002E with and without *A. brasilense* and different nitrogen fertilization rates (kg ha^−1^). Different capital letters indicate differences between the factor without and with inoculation, and different lowercase letters indicate differences between nitrogen rates within the factors without and with inoculation.

**Figure 4:**
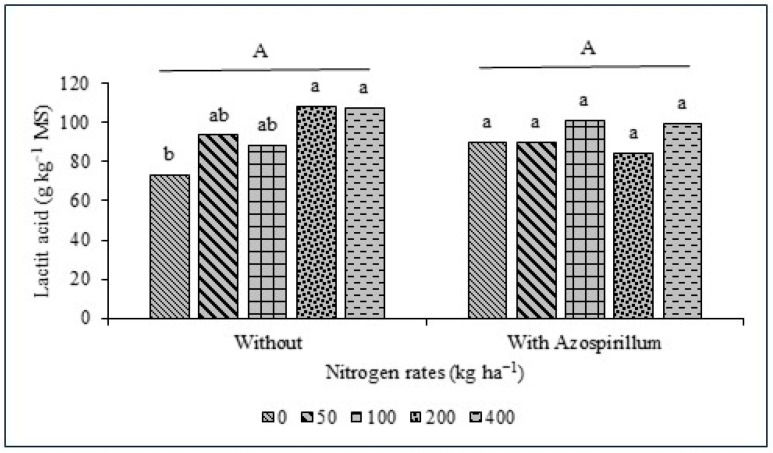
Average lactic acid values of AGRI 002E forage sorghum silage with and without inoculation with *A. brasilense* and different nitrogen fertilizer rates (kg ha^−1^). Different capital letters indicate the difference between the factor with and without inoculation, and different lowercase letters indicate the difference between the nitrogen rates within the factors with and without inoculation.

For the fermentation parameters of giant sorghum silage (Table 4), an interaction effect (*p* < 0.05) was observed between inoculation with *A. brasilense* and N rates for the N-NH_3_ (Figure 3) and lactic acid (Figure 4) contents.

Nitrogen fertilization promoted the lowest concentrations (*p* < 0.05) of acetic acid (ACE) in giant sorghum silages with the application of 100 (24.30 g kg^−1^ of DM) and 400 kg ha^−1^ of N (29.15 g kg^−1^ of DM). Nitrogen fertilization influenced (*p* < 0.05) the concentration of butyric acid (BUT), in which the rates of 100 and 200 kg ha^−1^ of N promoted lower values of butyric acid, being 5.12 and 4.64 g kg^−1^ of DM, respectively.

## 4. Discussion

This study tested the hypothesis that inoculation with *Azospirillum brasilense* strains AbV5 and AbV6 could partially supply the nitrogen (N) requirements of giant sorghum. However, the results showed that inoculation did not improve agronomic performance and reduced yield at higher N rates, whereas mineral N fertilization was the main driver of productivity. Although inoculation slightly increased silage crude protein concentration, this effect was insufficient to compensate for yield losses or to reduce the need for mineral N. Nitrogen fertilization also influenced silage composition, digestibility, and fermentation profile, reinforcing that optimal yield and silage quality remained strongly dependent on adequate N supply under the conditions of this study.

The significant increase in plant height, a direct result of nitrogen topdressing, underscores the potential of this nutrient to stimulate cell expansion and division, as well as the photosynthetic process. This promising result bodes well for the future of Bolivian giant sorghum, a high-yielding forage, as it indicates that nitrogen fertilization can boost plant height, thereby increasing vegetative input and, consequently, yield.

The increase in stem diameter of sorghum plants, while beneficial for the crop in terms of resistance to lodging and nutrient storage [46], presents a potential trade-off. Thicker stems, while more resistant, tend to be more lignified and dense, which can reduce fiber digestibility. This trade-off underscores the complexity of agronomic decisions and the critical need for a balanced approach to crop management.

During the experimental trial, plots fertilized with the highest N rates (200 and 400 kg ha^−1^ N) and sown with seeds inoculated with *Azospirillum* showed a higher incidence of aphid (*Melanaphis sacchari*) infestation. Pest control was therefore performed using imidacloprid (300 g ha^−1^ of active ingredient). After imidacloprid application, lodging of giant sorghum plants was observed, which may explain the lower yield recorded at the highest N rates and, consequently, the reduction in total yield.

In addition to aphid infestation, another probable cause of the lower yield observed in the first cycle may be associated with the inactivation of the nitrogenase enzyme complex, which regulates the activity of nitrogen-fixing bacteria in converting atmospheric N into ammonia, as described by Threatt and Rees [47]. This complex can be reversibly inhibited by the presence of ammonium ions, with its activity being restored once the added ammonium is consumed by bacterial metabolism [48].

The lower yield observed in the first cutting also cannot be ruled out as being partially associated with the presence of a native soil population of *Azospirillum* spp., although this aspect was not assessed in the present study. Such native populations may have reduced the plant response to bacterial inoculation. In soils already hosting diazotrophic microorganisms, competitive interactions and regulatory processes—such as a potential downregulation of nitrogenase activity—may limit the additional contribution of inoculation. These considerations highlight the importance of accounting for native soil microbial communities and their functional dynamics when interpreting yield responses, as reported by Döbereiner [49].

The most significant increases in silage dry matter observed in the study may be attributed to the action of N, which influences plant metabolism and growth, thereby increasing vegetative mass production [50,51] and N content in sorghum [52].

Nitrogen is a nutrient that plants require in large quantities; its deficiency is observed in most soils, and its supply in adequate amounts, in addition to increasing leaf area, increases chlorophyll content in the leaves, making them more efficient in intercepting solar radiation [53,54], and consequently, increasing dry matter accumulation. In general, the dry matter contents found in this study were within the recommendations for good-quality silage, ranging from 28% to 35% DM [55,56].

The observed values of neutral detergent fiber and acid detergent fiber in the silages were due to the low or absent grain content of the giant sorghum hybrid, since the grains contain little fiber, and the proportion of fiber present in the silage is directly correlated with the neutral detergent fiber content [57].

It is well known that higher levels of total digestible nutrients are desirable in silages; however, fibrous fractions tend to have lower energy availability [58]. This finding is supported by the present study, which revealed high levels of neutral detergent fiber, as well as lower in vitro digestibility of neutral detergent insoluble fiber [59]. The low or even absent panicle content in tall sorghum contributes to the low values of total digestible nutrients [60].

Although inoculation increased the NDF and ADF concentration of the silages, this change did not translate into differences in vitro digestibility of neutral detergent fiber (IVNDFD). NDF digestibility is mainly determined by cell wall structure, particularly lignin concentration and organization, which regulate the rate and extent of ruminal fiber degradation and influence intake and animal performance [61,62]. According to Neumann et al. [63], higher nitrogen fertilization increases the production of fibrous compounds in plants, reducing their digestibility. Van Soest [38] attributes nitrogen fertilization to reduced digestibility in forage plants, linking this to increased nitrogen compounds, increased cell wall compounds, and reduced water-soluble carbohydrates.

Similar results have been reported in forage grasses, indicating limited sensitivity of NDF digestibility to these management practices and strong dependence on genotype, environment, and harvest management [64,65]. Although ensiling may promote partial hemicellulose solubilization, these changes are generally insufficient to modify IVNDFD [66]. Therefore, *A. brasilense* inoculation and nitrogen fertilization, at the evaluated rates, did not affect ruminal fiber digestibility of sorghum silage, confirming that this trait is primarily governed by intrinsic plant characteristics and harvest maturity.

Silage pH remained within the optimal range (3.8–4.2) across all treatments, indicating efficient fermentation primarily driven by adequate dry matter content at ensiling, with minimal influence of *A. brasilense* inoculation or nitrogen fertilization [66,67]. This pH stability reflects the predominant role of harvest maturity and forage dry matter concentration in determining silage fermentation quality and likely contributed to limiting proteolytic activity, as evidenced by the consistently low ammonia nitrogen (N–NH_3_) concentrations. In well-fermented silages, N–NH_3_ values below 10% of total N indicate restricted protein degradation and adequate fermentation control [68]. In the present study, N–NH_3_ concentrations remained below 0.99% of total N in all treatments. Although treatments without inoculation generally showed lower N–NH_3_ concentrations, the interaction between *A. brasilense* inoculation and N rates maintained this parameter within recommended limits, indicating that inoculation did not compromise fermentative quality.

Lactic acid was the predominant organic acid in all silages, confirming the dominance of homolactic fermentation pathways, as expected for well-preserved silage [69]. Lactic acid concentrations exceeded those reported by Pinedo et al. [70] for forage sorghum silages and were within or above the range considered optimal for well-preserved silages (80–100 g kg^−1^ DM) [71]. The interaction between inoculation and N rates affected lactic acid concentration, suggesting that N availability modulated the fermentative pattern, possibly by influencing substrate availability and microbial activity during ensiling.

Acetic acid concentrations remained within the recommended threshold (≤2.5% of DM) [72] at the 100 kg ha^−1^ N rate, indicating a favorable fermentative profile. In contrast, silages produced without N fertilization exhibited markedly higher acetic acid concentrations (87 g kg^−1^ DM), which are associated with reduced palatability and voluntary intake, despite potential gains in aerobic stability [67,72,73]. Nitrogen fertilization likely favored a more efficient lactic fermentation by limiting heterofermentative pathways linked to acetic acid production. Butyric acid concentrations were reduced by N fertilization but remained above the recommended threshold (≤2.0 g kg^−1^ DM) [74], indicating partial suppression of clostridial activity. Inoculation had no effect on butyric acid concentration. According to Wang et al. [75], elevated butyric acid levels reflect the activity of *Clostridium* spp., which degrade proteins into ammonia and negatively affect silage quality.

## 5. Conclusions

Inoculation with *Azospirillum brasilense* strains AbV5 and AbV6 reduced first-cut yield at higher N rates and resulted in a lower total yield when averaged across the two harvests. Nitrogen fertilization remained the primary driver of biomass production, with the 200 kg ha^−1^ N rate promoting the highest yields. Both inoculation and N rates influenced selected silage fermentation parameters; however, all treatments produced adequately fermented silage. Nitrogen fertilization played a dominant role in increasing crude protein concentration, whereas inoculation contributed modestly to protein enrichment and was associated with higher neutral and acid detergent fiber fractions.

From a management perspective, these findings indicate that *A. brasilense* inoculation cannot substitute for mineral N fertilization in giant sorghum cultivation and should be applied with caution, particularly under higher N input conditions where yield reductions were observed. Under the conditions of this study, mineral N fertilization remains essential to achieve optimal productivity and silage quality. Future research should investigate the mechanisms underlying the negative yield responses to inoculation, including interactions with native soil microbiota and N availability, as well as alternative inoculation strategies and management practices.

## Figures and Tables

**Table 1:** Plant height (PH), stem diameter (SD), first cycle yield (FYield), regrowth yield (RYield), and total yield (Yield) of AGRI 002E sorghum silage with and without *A. brasilense* at different nitrogen rates.

Variables	*A. brasilense*		N Rates (kg ha^−1^)					SEM	*p*-Value		
	Without	With	0	50	100	200	400		A	N	A × N
PH (m)	3.22	3.12	2.96 b	3.26 ab	3.24 ab	3.30 a	3.08 ab	0.108	0.161	0.021	0.204
SD (cm)	1.39	1.33	1.20 b	1.39 a	1.41 a	1.43 a	1.37 a	0.045	0.050	0.001	0.408
FYield (t ha^−1^)	17.99	15.50	14.37	18.53	17.00	17.94	15.88	1.268	0.004	0.021	0.013
RYield (t ha^−1^)	4.13	3.73	3.12	3.50	4.38	4.71	3.95	0.655	0.346	0.137	0.917
Yield (t ha^−1^)	22.12 a	19.24 b	17.50 b	22.03 ab	21.38 ab	22.65 a	19.84 ab	1.566	0.007	0.020	0.074

Lowercase letters in the lines indicate differences within the nitrogen rates by Tukey’s test at 5% probability. A: *Azospirillum* effect; N: nitrogen rates effect; A × N: interaction effect between *Azospirillum* and nitrogen rates. SEM: Standard error of the mean.

**Table 2:** Dry matter (DM), crude protein (CP), neutral detergent fiber (NDF), and acid detergent fiber (ADF) of forage sorghum AGRI 002E without or with *A. brasilense* at different nitrogen rates.

	*A. brasilense*		N Rates (kg ha^−1^)					SEM	*p*-Value		
	Without	With	0	50	100	200	400		A	N	A × N
DM (%)	33.13	33.33	31.33	34.14	34.20	32.62	33.88	1.2310	0.8051	0.1173	0.1162
CP (%)	6.79	7.13	4.44 c	6.40 b	7.08 ab	8.12 ab	8.76 a	0.5897	0.3791	<0.0001	0.4642
NDF (%)	62.86	63.95	63.11	61.77	65.11	63.92	63.11	2.7437	0.5346	0.8063	0.6835
ADF (%)	39.64	39.95	39.90	38.53	41.34	39.78	39.41	2.0663	0.8099	0.7456	0.7418

Lowercase letters in the lines indicate differences within the nitrogen rates by Tukey’s test at 5% probability. A: *Azospirillum* effect; N: nitrogen rates effect; A × N: interaction effect between *Azospirillum* and nitrogen rates. SEM: Standard error of the mean.

**Table 3:** Dry matter (DM), crude protein (CP), neutral detergent fiber (NDF), acid detergent fiber (ADF), total digestible nutrients (TDN), in vitro digestibility of dry matter (IVDMD), and in vitro digestibility of neutral detergent insoluble fiber (IVNDFD) of AGRI 002E forage sorghum silage without or with *A. brasilense* at different nitrogen rates.

Variables	*A. brasilense*		N Rates (kg ha^−1^)					SEM	*p*-Value		
	Without	With	0	50	100	200	400		A	N	A × N
DM (%)	31.15	30.91	28.57 b	31.85 ab	32.09 a	31.41 ab	31.26 ab	0.986	0.708	0.009	0.183
CP (%)	7.01 b	7.67 a	5.39 c	6.52 b	7.22 b	8.39 a	9.19 a	0.3727	0.009	0.000	0.317
NDF (%)	68.20 b	70.79 a	71.13	68.88	70.00	68.30	69.14	1.8769	0.03	0.605	0.946
ADF (%)	44.06 b	46.54 a	45.74	44.55	46.58	44.71	44.92	1.7006	0.029	0.738	0.738
TDN (%)	60.09	59.87	59.91	60.87	58.90	59.99	60.25	1.4464	0.809	0.745	0.741
IVDMD (g kg^−1^)	595.50	594.83	612.32 ab	610.81 ab	622.38 a	582.54 ab	547.77 b	22.955	0.963	0.020	0.391
IVNDFD (g kg^−1^)	391.29	377.69	392.20	377.34	358.06	393.28	401.59	22.439	0.346	0.345	0.333

Lowercase letters in the lines indicate differences within the inoculant or in the nitrogen rates by Tukey’s test at 5% probability. A: *Azospirillum* effect; N: nitrogen rates effect; A × N: interaction effect between *Azospirillum* and nitrogen rates. SEM: Standard error of the mean.

**Table 4:** Hydrogen potential (pH), ammoniacal nitrogen (N-NH_3_), lactic acid (LAC), acetic acid (ACE), and butyric acid (BUT) of forage sorghum silage AGRI 002E with and without *A. brasilense* at different nitrogen rates.

Variables	*A. brasilense*		N Rates (kg ha^−1^)					SEM	*p*-Value		
	Without	With	0	50	100	200	400		A	N	A × N
pH	4.19	4.21	4.25	4.27	4.14	4.13	4.21	0.05	0.415	0.066	0.077
N-NH_3_	0.38	0.76	0.53	0.52	0.56	0.59	0.67	0.06	0.001	0.200	0.028
LAC	94.03	92.96	81.55	91.77	94.54	96.29	103.33	6.75	0.805	0.048	0.035
ACE	49.17	49.68	87.00 a	67.22 ab	24.30 b	39.46 ab	29.15 b	16.42	0.960	0.002	0.423
BUT	7.37	6.46	9.84 a	8.56 ab	5.12 bc	4.64 c	6.42 abc	1.27	0.265	0.001	0.443

Lowercase letters in the lines indicate differences within the nitrogen rates by Tukey’s test at 5% probability. A: *Azospirillum* effect; N: nitrogen rates effect; A × N: interaction effect between *Azospirillum* and nitrogen rates. SEM: Standard error of the mean.

## Data Availability

The data presented in this study are available upon request from the corresponding author.

## Abbreviations

The following abbreviations are used in this manuscript:

N	Nitrogen
PH	Plant height
SD	Stem diameter
FiYield	First-cycle yield
ReYield	Regrowth yield
Yield	Total yield
N-NH_3_	Ammonia nitrogen
DM	Dry matter
MM	Mineral matter
CP	Crude protein
NDF	Neutral detergent fiber
ADF	Acid detergent fiber
TDN	Total digestible nutrient
IVDMD	in vitro dry matter digestibility
IVNDFD	in vitro neutral detergent fiber digestibility
ACE	Acetic acid
BUT	Butyric acid
